# Use of a Flexible Inflatable Multi-Channel Applicator for Vaginal Brachytherapy in the Management of Gynecologic Cancer

**DOI:** 10.3389/fonc.2015.00201

**Published:** 2015-09-14

**Authors:** Samuel M. Shin, Tamara L. Duckworth, Benjamin T. Cooper, John P. Curtin, Peter B. Schiff, J. Keith DeWyngaert, Stella C. Lymberis

**Affiliations:** ^1^Department of Radiation Oncology, NYU Langone Medical Center, NYU Perlmutter Cancer Center, New York, NY, USA; ^2^Department of Obstetrics and Gynecology, NYU Langone Medical Center, New York, NY, USA

**Keywords:** multi-channel vaginal applicator, interstitial implant, single-channel applicator, toxicity

## Abstract

**Introduction:**

Evaluate use of novel multi-channel applicator (MC) Capri™ to improve vaginal disease coverage achievable by single-channel applicator (SC) and comparable to Syed plan simulation.

**Materials and methods:**

Twenty-eight plans were evaluated from four patients with primary or recurrent gynecologic cancer in the vagina. Each received whole pelvis radiation, followed by three weekly treatments using HDR brachytherapy with a 13-channel MC. Upper vagina was treated to 5 mm depth to 1500 cGy/3 fractions with a simultaneous integrated boost totaling 2100 cGy/3 fractions to tumor. Modeling of SC and Syed plans was performed using MC scans for each patient. Dosimetry for MC and SC plans was evaluated for PTV700 cGy coverage, maximum dose to 2 cm^3^ to bladder, rectum, as well as mucosal surface points. Dosimetry for Syed plans was calculated for PTV700 cGy coverage. Patients were followed for treatment response and toxicity.

**Results:**

Dosimetric analysis between MC and SC plans demonstrated increased tumor coverage (PTV700 cGy), with decreased rectal, bladder, and contralateral vaginal mucosa dose in favor of MC. These differences were significant (*p* < 0.05). Comparison of MC and Syed plans demonstrated increased tumor coverage in favor of Syed plans which were not significant (*p* = 0.71). Patients treated with MC had no cancer recurrence or ≥grade 3 toxicity.

**Conclusion:**

Use of MC was efficacious and safe, providing superior coverage of tumor volumes ≤1 cm depth compared to SC and comparable to Syed implant. MC avoids excess dose to surrounding organs compared to SC, and potentially less morbidity than Syed implants. For tumors extending ≤1 cm depth, use of MC represents an alternative to an interstitial implant.

## Introduction

Patients with recurrent endometrial cancer following hysterectomy or primary vaginal cancer with disease limited to the pelvis are often treated with multi-modality treatment with curative intent. One option includes the use of whole pelvis radiation followed by either a tumor directed boost using brachytherapy or focused external beam radiation to disease in the vagina (1–6). The type of brachytherapy used is dependent on depth and size of tumor within the vaginal vault. Non-bulky disease, ≤5 mm in thickness or depth, can be treated with intracavitary brachytherapy, while bulky disease, >5 mm in thickness or disease beyond the vaginal surface, is more suitable for an interstitial approach (3). Patients presenting with extensive vaginal or paravaginal disease, disease with bulky parametrial extension, disease in the vaginal apex, or pelvic anatomy resulting in suboptimal dose distribution using intracavitary brachytherapy may also benefit from treatment using an interstitial implant (7–10).

Although non-bulky disease in the vagina or vaginal cuff can be treated with a rigid intracavitary single-channel vaginal cylinder, there is no ability to sculpt dose away from organs at risk (OARs), such as the bladder and rectum. Multi-channel applicators (MCs), however, can allow for preferential treatment of lateralized disease while decreasing dose delivered to OARs through a modulation of dwell times in various positions along the channels (11). In addition, dose gradient in the radial direction allows steep dose falloff as source channels are placed close to the tumor volume compared to a single-channel applicator (SC) (12). Furthermore, with preferential dwell times, sparing of OARs distant to the lesion being treated can be spared due to the radial dose inhomogeneity achieved by strategic channel loading.

Despite a study demonstrating superior dosimetry between MC and SC with respect to tumor coverage and decreased dose to OARs, SC are more commonly used due to decreased cost, increased ease of use, and availability (11, 13). One recent dosimetric study compared use of a MC with that of a SC and demonstrated similar tumor coverage, however with statistically significant dose reduction to bladder and rectum in favor of the MC when compared to a SC at 5 mm prescription depth (13).

**Figure 1:**
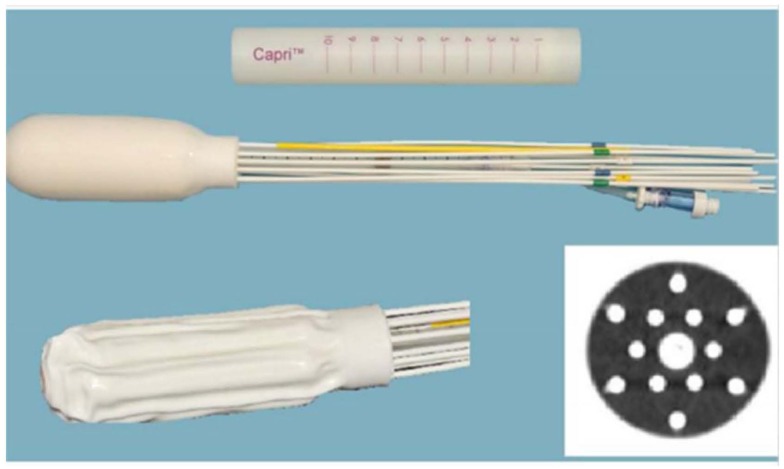
**Multi channel (Capri) applicator inflated and deflated with display of axial cross-section**.

The first clinical implementation of the newly FDA approved Capri™ multi-channel applicator in five patients demonstrated reduced rectal dose and suggested usefulness to cover extensive vaginal disease (14). In our initial experience, we report the use of the Capri™ multi-channel applicator in the treatment of vaginal disease originating from primary vaginal or endometrial cancer (Figure 1). Compared with the published experience using the Miami Multichannel Applicator where a plan was generated prior to the first treatment and used for all fractions, patients underwent real-time planning prior to all three fractions (15). In addition, we compared dosimetry of MC to a SC and an interstitial implant. The goals of our study are first to compare dose distributions representative of MC, SC, and interstitial implants under conditions where tumor extends up to 1 cm depth and second to evaluate our clinical experience using the MC with respect to efficacy and toxicity of treatment. Dosimetry was compared between a MC and a SC with respect to tumor coverage and dose to OARs, and between a MC and an interstitial implant (Syed) with respect to tumor coverage.

## Materials and Methods

Four patients with diagnosis of primary vaginal cancer (*n* = 2) or recurrent endometrial cancer (*n* = 2) with disease limited to the vagina were treated with a MC between December 2012 and June 2013. The MC used in this study was the Capri™(Varian Medical Systems, Palo Alto, CA, USA) applicator, a single use, flexible, inflatable (2011 US Food and Drug Administration-cleared) vaginal applicator with 13 channels (Figure 1). The applicator consists of a central channel, an inner array of six channels, and an outer array of six channels. Each lumen is impregnated with barium sulfate in addition to internal markers allowing for identification of each catheter. The applicator has a deflated insertion diameter of 29 mm, but is inflated using saline to conform to a patient’s pelvic anatomy.

Prior to treatment with MC, all patients underwent appropriate staging evaluation with pelvic examination, biopsy, and imaging, including computerized tomography (CT) and/or magnetic resonance imaging (MRI) of the abdomen and pelvis prior to consultation. During pelvic examination, patients had up to two fiducial markers placed into the submucosa near the site of disease in the vagina. All fiducial markers were placed by a single surgeon. Following fiducial marker placement, patients were treated with whole pelvis radiation to a dose of 4500 cGy in 25 fractions using a four-field technique with photons. Following whole pelvis treatment, patients underwent pelvic examination to determine response of disease in the vagina. All four patients presented with tumor >5 mm, but ≤10 mm in depth and size at the time of pelvic re-examination, and were selected to be treated with the MC instead of a SC or Syed.

Patients were instructed to drink 16 ounces of water prior to insertion of the MC. A foley catheter was not placed prior to insertion of MC. Local anesthetic gel and lubricant gel was utilized prior to inserting the deflated MC into the patient’s vagina. Following insertion, the MC was inflated to the largest diameter with saline to the shape and diameter of the patient’s vaginal anatomy to prevent displacement of the applicator, to reduce air gaps (16), to reduce vaginal mucosal dose, thereby achieving optimal dose distribution. A 1.25 mm axial CT slice thickness CT scan was generated at 120 kVp using a LightSpeed RT 16 CT Simulator (GE Healthcare, Waukesha, WI, USA). The CT images were transferred to the Eclipse treatment planning system (Varian, Palo Alto, CA, USA). One physician contoured the bladder, rectum, and the gross tumor volume (GTV), representing vaginal tumor demarcated by fiducial markers. Rectum was contoured from the recto-sigmoid junction superiorly to the ischial tuberosity inferiorly, and the entire bladder was contoured. Physicists then contoured the MC, the PTV500 cGy (planning target volume to receive 500 cGy) and the PTV700 cGy (planning target volume to receive 700 cGy). The PTV500 cGy was created with a 5 mm expansion of the MC and a 3 mm expansion of the GTV combined as a Boolean structure, while the PTV700 cGy was defined as equivalent to the GTV. OARs (bladder and rectum) were not cropped too closely to mimic traditional cylinder planning and to account for bladder and rectal filling changes between the time of CT and the time of treatment plan delivery.

**Figure 2:**
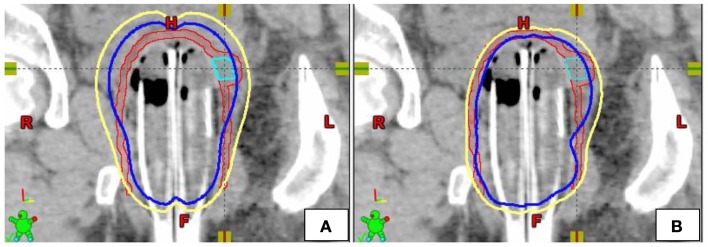
**Example of single-channel (A) and multi-channel (B) dosimetric plans in the coronal plane [yellow = 5 Gy isodose line, blue = 7 Gy isodose line, Red = PTV5Gy, Teal = PTV7Gy (GTV)]**.

**Figure 3:**
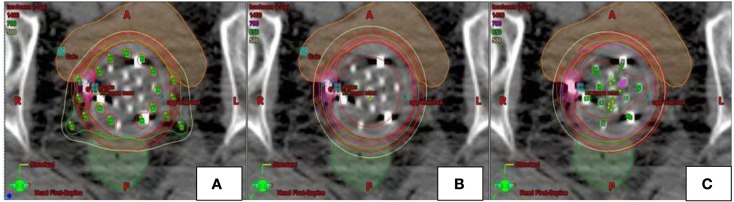
**Example of dosimetric plans [Syed (A), single-channel (B), multi-channel (C)] for a patient with recurrent endometrial cancer in the axial plane**. Multi channel plan **(C)** was created using 13-channel applicator. SC **(B)** and Syed **(A)** plans modeled using pre-existing MC plan. Location of catheters loaded for each of the plans is depicted in green.

Plans were created with the MC in place, and each patient received three weekly insertions of Iridium-192 high dose rate MC treatment (Figures 2B and 3C). A CT simulation was performed for each fraction (weekly insertion), and a separate plan was created from the CT scan. A total dose of 15 Gy was delivered to the upper two-thirds of the vagina, prescribed to a depth of 5 mm in three weekly fractions with a simultaneous integrated boost (SIB) of 21 Gy to the PTV700 cGy in three weekly fractions, prescribed to a depth up to 10 mm from the applicator surface. Varian Brachyvision was used for treatment planning with source dwell positions separated by 5 mm (Varian, Palo Alto, CA, USA). Volumetric optimization was used, setting appropriate dose constraints to deliver the prescribed doses to the target volumes.

Contoured OARs were utilized to help conform the dose to target volumes. Post-optimization, each plan was evaluated and modified to eliminate the use of catheters with very few, low dwell time source positions. Outer catheter use was limited to the coverage of the PTV700 cGy when possible. Dosimetry for MC plans was evaluated for PTV coverage, maximum D2cm^3^ to bladder and rectum, as well as vaginal mucosal surface points ipsilateral and contralateral to PTV700 cGy. An α/β = 3 was used to determine cumulative biological effective dose BED_3_ (Gy) of whole pelvis radiation and three weekly insertions of MC to the normal structures bladder, rectum, and including proximal vagina. An EQD2 (converting the BED_3_ values to equivalent total doses delivered in 2 Gy fractions) was also calculated for dose to vagina. Following treatment with the MC, patients were seen in follow-up at 1, 3, 6 months, and yearly thereafter.

Modeling of SC plans was performed using existing initial planning CT data sets for all four patients treated with the MC (Figures 2 and 3). The SC plans were modeled by loading the single central catheter of the MC. SC plans were optimized for PTV700 cGy coverage, maximum D2cm^3^ to bladder and rectum as well as mucosal surface points. In order to ensure fair comparison, the D95 of PTV500 cGy was set equivalent for each of the MC and SC plans. Syed plans were also generated using existing initial planning CT data sets for all four patients treated with the MC (Figure 3), and optimized for PTV700 cGy coverage. One Syed plan was created for each treated patient to simulate a single interstitial insertion used according to our institutional practice. We measured dose to the tumor and vagina, but did not measure dose to the OARs (bladder and rectum) as we would avoid inserting interstitial needles into these surrounding organs.

A Wilcoxon signed rank sum test for non-parametric data was used to compare various dosimetric points of MC versus SC, and MC versus Syed, with significance assessed at a *p* < 0.05. All statistical analyses were performed using SPSS version 21 (SPSS Inc., Chicago, IL, USA). Following treatment with MC, all patients were evaluated for tumor response and bladder, rectal, and vaginal toxicity assessed with the radiation therapy oncology group (RTOG) acute and late radiation morbidity scoring system. This retrospective study was approved by the institutional review board.

## Results

A total of 28 plans, including 12 MC plans, 12 SC plans, and 4 Syed plans were generated in this study. The treated length along the MC measured an average of 6.6 cm (range, 5.7–7.9 cm) and the average number of loaded catheters used in each optimized plan was 11 (range, 9–13 catheters). The applicator was inflated with saline to a median volume of 72.5 cm^3^ (range, 45–87 cm^3^). Unlike a fixed diameter rigid SC, the diameter of the MC varied according to the patient’s pelvic anatomy. The average diameter 10 mm from apex of the MC and the maximum diameter of the MC measured 36.0 mm (range, 27.6–42.0 mm) and 44.0 mm (range, 33.1–50.0 mm), respectively. Median planning time, from completion of CT simulation with MC in place to completion of plan, was 140.5 min (range, 73–188 min). Median time for MC procedure from simulation to removal of applicator was 3 h. Patients who had discomfort with bladder distention were given narcotic medications. All patients were treated without a foley catheter and underwent MC treatment with a full bladder.

The PTV700 cGy for the 12 MC plans measured an average volume of 2.0 cm^3^ (range, 1.6–3.1 cm^3^) at an average depth of 9.0 mm (range, 7.0–9.7 mm) from the vaginal surface. Target coverage for patients treated with the MC was optimized to a mean D99 and D95 of 711.6 cGy (range, 679.5–747.6 cGy) and 749.3 cGy (range, 722.0–782.2 cGy), respectively. In addition to targeting PTV700 cGy with a SIB to a planned dose of 700 cGy, the upper vagina was treated to a planned dose of 500 cGy at 5 mm depth. The mean D99 and D95 to the upper vagina (PTV500 cGy) were 484.0 cGy (range, 400.7–561.7 cGy) and 523.3 cGy (range, 472.9–610.8 cGy), respectively. The mean D2cm^3^ to bladder and rectum were 576.4 cGy (range, 407.4–717.8 cGy) and 622.8 cGy (range, 548.1–713.5 cGy), the mean ipsilateral vaginal mucosa point dose within PTV700 cGy was 1448.2 cGy (range, 941.8–2137.8 cGy), while the mean contralateral vaginal mucosa point dose contralateral to PTV700 cGy was 882.0 cGy (range, 659.7–1384.7 cGy). The median maximum cumulative BED_3_ to the proximal vaginal mucosa in the area of tumor was 131.8 Gy_3_ (range, 119.5–136.7 Gy_3_), and median maximum cumulative EQD2 Gy_3_ to the proximal vaginal mucosa in the area of tumor was 206.6 Gy (range, 151.8–237.9 Gy). Contralateral median cumulative dose to vaginal mucosa was 107.7 Gy_3_ (range, 104.8–109.0 Gy_3_), corresponding to a median cumulative EQD2 of 64.6 Gy (range, 62.9–65.4 Gy).

Modeling of 12 SC plans was created by setting the D95 of PTV500 cGy equivalent between the 12 MC and 12 SC plans. Mean D99 and D95 of PTV700 cGy were 643.2 cGy (range, 486.68–682.47 cGy) and 674.1 cGy (range, 475.37–706.43 cGy), respectively. There was a 10.6 and 11.2% decrease in tumor coverage, respectively, compared to MC plans. Improvement in dose coverage of PTV700 cGy was statistically significant (*p* < 0.05) in favor of the MC plans compared to the SC plans. Mean ipsilateral vaginal mucosa point dose in the SC plans was 1167.7 cGy (range, 910.9–1428.9 cGy). Mean D2cm^3^ to bladder, rectum, and contralateral vaginal mucosal point dose were 707.1 cGy (range, 536.52–1005.33 Gy), 706.0 cGy (range, 638.97–746.3 cGy), and 1166.9 cGy (range, 935.3–1404.0 cGy), respectively, with a 22.7, 13.4, and a 32.3% increase in dose, respectively, when compared to MC plans (Table 1). Decreased dose to surrounding OARs was statistically significant (*p* < 0.05) in favor of MC compared to SC plans.

**Table 1:** **Comparison of dosimetry to target (PTV) and dose to organs at risk between multi-channel and single-channel applicator**.

	PTV700 cGy D99 (cGy)	PTV700 cGy D95 (cGy)	PTV500 cGy D99 (cGy)	PTV500 cGy D95 (cGy)	D2cc bladder (cGy)	D2cc rectum (cGy)	Contralateral mucosa (cGy)	Ipsilateral mucosa (cGy)
MC	711.6	749.3	484.0	523.3	576.4	622.8	882.0	1448.0
SC	643.2	674.1	476.8	524.2	707.1	706.0	1167.0	1168.0
% change (MC/SC)	+10.6	+10.4	+1.5	−0.2	−18.5	−11.8	−24.4	+23.9
*p-*Value*	0.005	0.003	0.084	0.433	0.008	0.006	0.023	0.002

*MC, multi-channel applicator; SC, single-channel applicator*.

*Using Wilcoxon signed rank sum test.

Modeling of four Syed plans were also performed by maintaining equivalence between the D95 of PTV500 cGy between four MC and four Syed plans. Optimization required use of 15 catheters for each modeled Syed plan. Syed mean D99 and D95 of PTV700 cGy was 717.0 cGy (range, 699.0–747.4 cGy) and 739.2 cGy (range, 716.4–774.3 cGy), with a 0.8% increase and 1.3% decrease in dose, respectively, when compared to MC plans (Table 2). However, tumor coverage (PTV700 cGy) was not statistically significant when comparing the Syed to MC plans. An example of the range of dosimetric values for a patient planned with a MC, SC, and Syed is shown in Table 3.

**Table 2:** **Comparison of dosimetry to target (PTV) between multi-channel applicator and Syed**.

	PTV700 cGy D99 (cGy)	PTV700 cGy D95 (cGy)	PTV500 cGy D99 (cGy)	PTV500 cGy D95 (cGy)
MC	711.6	743.9	484.0	523.3
Syed	717.0	739.2	486.8	539.9
% change (MC/Syed)	−0.8	0.6	−0.6	−3.1
*p-*Value*	0.273	0.715	0.715	0.068

*MC, multi-channel applicator*.

*Using Wilcoxon signed rank sum test.

**Table 3:** **Dosimetry to target (PTV) and OARs in example patient shown in Figure 3 using multi-channel applicator, single-channel applicator, and Syed**.

	PTV 700 cGy D99	PTV 700 cGy D95	PTV 500 cGy D99	PTV 500 cGy D95	D2cc bladder (cGy)	D2cc rectum (cGy)	Contralateral mucosa (cGy)	Ipsilateral mucosa (cGy)
**MC**
Insertion 1	720.2	743.6	469.8	497.0	644.2	635.7	1057.6	1478.3
Insertion 2	739.4	782.2	461.2	502.8	550.9	587.7	721.2	2137.8
Insertion 3	731.3	761.1	488.8	516.7	623.9	569.8	683.2	1235.2
**SC**
Insertion 1	637.0	656.6	466.6	498.2	743.3	694.6	1077.5	1258.6
Insertion 2	677.0	709.5	459.3	500.5	692.0	694.2	1058.7	1400.5
Insertion 3	677.9	704.2	465.2	515.1	729.4	825.4	1298.7	1144.7
**Syed**
Insertion 1	692.2	716.4	419.7	531.0	N/A	N/A	N/A	N/A

*MC, multi-channel applicator; SC, single-channel applicator; N/A, not applicable*.

At a median follow-up of 17.3 months (range, 12.4– 18.2 months), one patient presented with grade 2 gastrointestinal toxicity (rectal bleeding without medical intervention) which has since resolved at her most recent follow-up at 16.7 months, one presented with grade 2 vaginal toxicity (ecchymosis in the vaginal mucosa) at 12.4 months, and another presented with grade 1 vaginal toxicity at most recent follow-up at 18.2 months. Toxicity was minimal overall at each patient’s most recent follow-up (Table 4). In addition, there was no evidence of vaginal disease on pelvic examination or pelvic disease on imaging in all four patients treated with whole pelvis radiation followed by treatment with MC.

**Table 4:** **Record of toxicity following treatment with whole pelvis radiation and multi-channel applicator treatment using the RTOG Late Radiation Morbidity Scoring Schema**.

Patient	Follow-up (months)	Gastrointestinal toxicity	Bladder toxicity	Vaginal toxicity
1	12.4	None	None	Grade 2
2	18.2	None	None	Grade 1
3	16.7	Grade 2^a^	None	None
4	17.8	None	None	None

*^a^Grade 2 rectal bleeding noted at 1 year completely resolved at 1.5 years follow-up*.

## Discussion

We demonstrated that use of a MC such as the Capri™ provides superior dosimetric coverage of tumor volume with lower dose to surrounding normal tissues compared to a SC in the four cases studied. The results of this study, an 18.5 and 11.8% absolute reduction in the bladder and rectum D2cm^3^, are consistent with other series demonstrating a reduction in the bladder and rectum dose, in favor of a MC compared to a SC (13). In addition, MC provides comparable dosimetric coverage versus a Syed implant for vaginal tumors >5 mm but ≤10 mm in depth. Although a prior study has compared SC to MC, this dosimetric comparison between a MC and Syed has to our knowledge not been studied until now.

A potential criticism of this study is that all SC and Syed plans were generated using the patient’s MC-based CT data set. Although our study compared a MC with a SC, all SC plans were modeled with the patient’s existing MC CT data. As a result, the modeled SC cylinder diameter is larger than one would expect in a clinical setting where a rigid SC cylinder is used. This is due to intrinsic properties of the MC, as the diameter can be increased asymmetrically to sizes greater than is commercially available with SC. Although comparison of a MC to a SC was derived using a MC image data set, these results should translate to a situation using a CT-set with a rigid SC in place and would represent the best case scenario for the SC configuration. Future studies may include simulating patients with both a MC and a rigid SC cylinder to better compare differences in dosimetry with respect to the target volume and OARs.

Due to the increased complexity and longer planning time (median 140.5 min) using the Capri™ device compared to using a SC, a potential tradeoff using this device may be underdosing the tumor volume or exaggeration of the magnitude in dose reduction to OARs. However, re-planning prior to delivery of each fraction and use of fiducial markers in the area of the target volume were strategies used to help offset these potential errors. Increased familiarity and creating a streamlined approach using this device may further decrease any potential intra or interfractional errors.

Patients with vaginal tumors >10 mm in depth following whole pelvis radiation were not included in this study due to concerns of unacceptably high risks of toxicity to surrounding normal tissue including vagina, bladder, and rectum. Patients with vaginal tumors located in the apex of the vaginal cuff may lead to increased dose to overlying sigmoid colon or bowel when treated with MC prescribing to deeper coverage for tumors in this location. Both clinical situations involving deeper (>10 mm) or apical tumors may best be addressed using an interstitial implant with laparoscopic displacement of bowel if necessary.

Although it was possible to load fewer than 13 catheters when treating patients in this study, an increased number of catheters allowed flexibility in plan optimization with improved tumor coverage while reducing risk of treatment-related morbidity to the bladder, rectum, and vagina. The optimal number of catheters to use in a patient is highly dependent on a number of factors, including tumor location, depth of tumor, pelvic anatomy, and the ability to deliver a SIB.

Clinically, the Capri™ was rigidly secure in the patient after saline filling, demonstrating reliable positioning in the rotational and transverse planes. As shown in Figure 1, the apical yellow catheter demarcates zero degrees ensuring accurate placement and the central catheter was marked ensuring that the device was not slipping out of the patient. After planning, MC device positioning was clinically confirmed prior to proceeding with treatment. Replanning therefore was not deemed necessary. However, future evaluation may be performed to evaluate intrafraction variability.

Our patients were treated with a SIB approach to the PTV700 cGy demarcated by clips which depending on device placement could vary in position; therefore, we felt that replanning was necessary to be performed prior to each fraction delivered. SIB treatment allowed for contralateral vaginal mucosa sparing that may reduce long-term vaginal toxicity. In addition, our median cumulative proximal vaginal mucosal dose (EQD2) was within the reported threshold (EQD2 of 238 Gy) for proximal vaginal necrosis (17), with no events >grade 2 vaginal toxicity reported in our cohort.

Replanning with the Capri™ device may not be necessary in the interfraction setting if one utilizes uniform dose prescription to the vaginal target. As demonstrated by a dosimetric study, replanning was not necessary prior to each fraction using SC or MC vaginal cylinders with respect to OAR (18).

Despite these limitations, a MC such as the Capri™ can serve as a viable non-invasive treatment modality alternative to an interstitial implant for patients with vaginal tumors up to 10 mm in depth with preferred dosimetry, compared to SC which would produce unacceptably high normal tissue doses. At a median follow-up of 17.3 months, all patients treated with the MC presented with no evidence of tumor recurrence on pelvic exam with minimal treatment-related toxicity. Furthermore, use of a MC under these conditions can result in significant cost savings, more rapid recovery time and is a less invasive procedure compared to a traditional Syed applicator (10, 19–21). However, a larger cohort of patients and continued follow-up of treated patients are required to assess long-term clinical outcomes, including disease control and tissue toxicity using this technical approach.

## Author Contributions

All authors (SS, TD, BC, JC, PS, KD, and SL) have contributed to the conception, draft/revision, and approval of final draft and are accountable for all aspects of work in ensuring that questions related to accuracy or integrity of work are appropriately investigated and resolved.

## Conflict of Interest Statement

The authors declare that the research was conducted in the absence of any commercial or financial relationships that could be construed as a potential conflict of interest.

## Abbreviations

MC, multi-channel applicator; OAR, organs at risk; PTV500 cGy, planning target volume to receive 500 cGy; PTV700 cGy, planning target volume to receive 700 cGy; SC, single-channel applicator; SIB, simultaneous integrated boost.
